# Empirical Data-Driven Linear Model of a Swimming Robot Using the Complex Delay-Embedding DMD Technique

**DOI:** 10.3390/biomimetics10010060

**Published:** 2025-01-16

**Authors:** Mostafa Sayahkarajy, Hartmut Witte

**Affiliations:** Group of Biomechatronics, Fachgebiet Biomechatronik, Technische Universität Ilmenau, D-98693 Ilmenau, Germany; hartmut.witte@tu-ilmenau.de

**Keywords:** bio-inspired locomotion, soft robotics, bio-robotics, data-driven modeling, CDE DMD

## Abstract

Anguilliform locomotion, an efficient aquatic locomotion mode where the whole body is engaged in fluid–body interaction, contains sophisticated physics. We hypothesized that data-driven modeling techniques may extract models or patterns of the swimmers’ dynamics without implicitly measuring the hydrodynamic variables. This work proposes empirical kinematic control and data-driven modeling of a soft swimming robot. The robot comprises six serially connected segments that can individually bend with the segmental pneumatic artificial muscles. Kinematic equations and relations are proposed to measure the desired actuation to mimic anguilliform locomotion kinematics. The robot was tested experimentally and the position and velocities of spatially digitized points were collected using QualiSys^®^ Tracking Manager (QTM) 1.6.0.1. The collected data were analyzed offline, proposing a new complex variable delay-embedding dynamic mode decomposition (CDE DMD) algorithm that combines complex state filtering and time embedding to extract a linear approximate model. While the experimental results exhibited exotic curves in phase plane and time series, the analysis results showed that the proposed algorithm extracts linear and chaotic modes contributing to the data. It is concluded that the robot dynamics can be described by the linearized model interrupted by chaotic modes. The technique successfully extracts coherent modes from limited measurements and linearizes the system dynamics.

## 1. Introduction

The agility and energy efficiency of animals’ swimming have triggered roboticists and researchers to investigate bioinspired locomotion methods for the navigation of robotic systems within fluidic environments. Furthermore, miniature swimming robots [1,2] or soft robots [3] are designed to move based on biomimetic propulsion as the conventional propellers with electric motors are too bulky or heavy for such applications. Nevertheless, the aquatic locomotion of animals contains complex dynamics as a mirror of interacting neuromuscular activities and hydrodynamics. In anguilliform swimming, the entire body participates in the locomotion, which makes the body–fluid interaction sophisticated to simulate and analyze. Each part of the body contributes to propulsion, unlike in other forms of undulatory locomotion where only certain body parts might move. Anguilliform locomotion is generally slower than other types of swimming, such as in the other extreme of thunniform locomotion seen in tunas and sharks. However, it is highly energy efficient [4] and allows the animals to navigate complex environments like narrow crevices and dense vegetation (which is one of the main obstacles for technical drives with rotatory propulsion). The efficiency may be attributed to the phenomenon that a wake-induced drag vanishes while the vortex wake is absent [5]. The propeller drives for boats cannot be used in the overgrown edge zones of shallow waters, as the plants block the rotational movement very quickly.

The technical implementation of fish-like propulsion systems is an alternative to standard engineering solutions. Fish use muscle fibers to bend their bodies. These muscle fibers actively generate tension on the concave side while passively extending on the convex side. The most analogous artificial muscle is the McKibben actuator [6], a lightweight and flexible actuator well-suited for soft swimming robots. In [7], it is discussed that the pneumatic soft actuators’ force generation characteristics are similar to the natural muscles. Furthermore, they are well-suited for underwater field applications because of the absence of metal components and operating with pneumatic actuation. Industrial pneumatic pressurization enables McKibben actuators to generate sufficient force to operate effectively, even in the high-density water environment.

In anguilliform swimming, three components including fluid dynamics, neural control, and the musculoskeletal system interact with each other. While studying the system as a whole is a formidable task, considerable research has been devoted to different aspects of anguilliform locomotion. This swimming mode is observed ubiquitously in single-cell organisms with tiny sizes up to lampreys and eels, covering Reynolds numbers on the order of 10^−3^ to 10^3^. Anguilliform locomotion involves complex undulations as a result of superimposing traveling mechanical waves, as well as stationary waves [8,9]. A complex traveling wave can be conceptualized as an interplay of the configurations defined by the real and imaginary components of the wave [10]. The real part becomes apparent when the temporal modulation of the imaginary part is zero, while the imaginary part emerges when the temporal modulation of the real part vanishes.

Video recording, followed by video processing and particle image velocimetry (PIV), seems to be the standard technique to calculate the kinematics of anguilliform swimming of animals like eels and lampreys [11,12]. The kinematic model can be used for hydrodynamic simulations to investigate the underlying physics and interaction forces. The bending kinematic creates vortices in the environment producing negative pressure regions that pull the fish forward with the suction trust according to [11]. Transsected animals are additional subjects for studying altered kinematics within the locomotion mode. In healthy lampreys, the amplitude of the traveling wave increases as it travels along the body, but spinal-transacted lampreys exhibit a uniform wave with less amplitude and wavelength [12]. Nevertheless, studying transected animals has some complexities and is subject to further problems such as decreased muscle force and complex neural responses. On the other hand, bio-inspired robots can serve as manageable devices for mimicking animal kinematics. Some eel-like robots have been developed by different robotic laboratories worldwide. Examples include AmphiBot I [13] and II [14,15].

However, the robots consisting of serially jointed rigid links lack the compliance of natural swimmers. Commonly, the gesture of conventional robots cannot passively be affected by external forces. On the other hand, emerging rapidly in the last decades, soft robotics promote compliant-body systems without explicit joints and rigid links of conventional robots. In recent years, researchers have developed some eel-like soft robots with pneumatic [16,17,18,19,20,21,22,23] or cable-driven actuation [24,25]. A soft robot is structurally an infinite dimensional system that deforms due to both actuator force and the interaction force exerted by the environment. Due to technology limitations, manipulating only a limited number of active degrees of freedom (DOFs) is conceivable in soft robots. This makes soft robots underactuated systems that may not outperform real fish. Nevertheless, a soft-body robot is qualitatively more similar to its natural counterpart, making them better candidates for either investigating the swimming principles or replicating the natural performance within robotics.

Our previous study, ref. [7], showed that complex body–fluid interaction produces different modes that appear as exotic fluctuations in the swimmers’ midline. The study employs numerically expensive fluid–solid interaction (FSI) simulations to extract the system modes. Extracting the rearward moving waves related to the anguilliform locomotion is generally difficult. Such decomposition and analysis are important for evaluating and modeling the locomotion of the robots, which are still far from the efficiency of their natural counterparts. Nevertheless, the FSI method has limitations, including modeling approximations and huge numerical costs. In this work, we investigate a method to analyze the experimentally measured data without any measurements or calculations of the fluid environment.

In the case of soft swimming robots, the generation of traveling waves is complex and comprises ostensibly noisy movements of the robot due to the softness of the robot’s structure. Coherent structures cannot be visually observable when multiple chaotic modes or additional parasitic and noisy signals influence the system states. Machine learning (ML) methods, and especially linearization methods based on dynamic mode decomposition (DMD) and its variants, can help analyze anguilliform locomotion. Showcase studies include examining various data from mathematically expressed sinusoidal traveling waves to videos taken from natural swimmers like eels in experimental water tanks. DMD, originally derived from Koopman analysis, proposes a linear model for data sequences [26] and is investigated to analyze fluid dynamics [27,28]. The method acts as a modal decomposition technique with a separation of temporal and spatial variables, providing a linearized model that may be used for estimation or control purposes. In [29], the method is used to linearize a worm robot model. The ability of DMD-based data-driven modeling methods to extract spatiotemporal patterns from data makes them ideal for analyzing traveling wave phenomena. When partial state information is available, delay coordinates can be a better choice of observables to approximate the Koopman operator in establishing a DMD model.

In this work, the swimming kinematics of a soft eel robot is analyzed using pure experimental data. A pneumatic eel robot and its actuation kinematic equations are introduced. The robot contains six segments that are individually actuated using contraction-type pneumatic actuators. The robot is experimentally tested and a data-driven algorithm is proposed to inspect coherent and linear patterns within the exotic fluctuations in the robot. First, in Section 2.1, the robot structure and the relations that relate actuator contraction to the traveling mode are presented as the working principle. Section 2.2 explains the complex delay-embedding DMD algorithm (CDE DMD). Delay embedding is used to make data-driven models from partial measurements to understand the dynamics of partially observed systems. The simulation results are presented in Section 3. In Section 4, it is discussed that the algorithm revealed linear and chaotic modes within the data, enabling us to partition the system into linear and nonlinear dynamics. The conclusion is summarized in Section 5.

## 2. Materials and Methods

### 2.1. Structure and Actuation Kinematics

**Figure 1:**
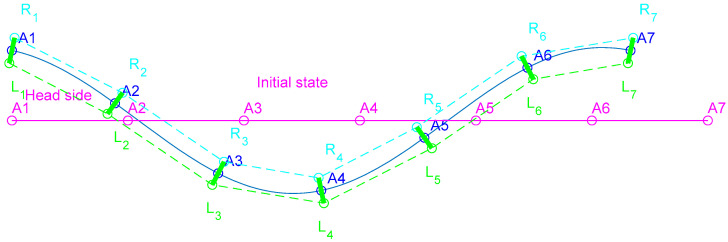
Geometrical model of the robotic fish. The solid lines represent the robot backbone midline, and the dashed lines show the actuators’ nominal axes.

The soft robot structure contains a flexible beam and seven rigid parts for connecting the actuators and balancing the robot on the water surface. Twelve pairs of contraction-type pneumatic muscles provide lateral bending for each segment of the tethered robot. In contrast with similar rigid robots in which actuation torque can be exerted on the rotational joints, the soft robot is driven by linear (i.e., axial) actuators. This brings more similarity with natural swimmers but requires measurement of the muscle actuation needed for a desired kinematic. Inspired by primitive natural swimming, the kinematic relations are derived with a supposition that the neutral axis of the robot in the steady state follows a moving sine wave. The schematic kinematic model of the robot is shown in Figure 1. The horizontal line represents the inactivated initial state of the robot backbone midline. Seven equally distanced points, *A*_1_, *A*_2_, … *A*_7_, represent the position of rigid connectors (also used as floats). The *R*_1_*L*_1_, *R*_2_*L*_2_, …, *R*_7_*L*_7_ are rigidly connected to the flexible backbone, and the dashed lines represent actuation axes. The actuation state vector $\vec{q}=\{q_{1},q_{2},q_{3},\ldots ,q_{12}\}$ is defined with the Euclidean distances $q_{i}\;i=1..12$.

(1) $$q_{i}=\left|R_{i}R_{i+1}\right|\;,\;q_{i+6}=\left|L_{i}L_{i+1}\right|\;i=1..6$$

Geometric relations are used to obtain the actuator lengths for the given desired deformation to generate a locomotion pattern. Supposing the desired deformation of the neutral axis is shown as:

(2) $$f(x,t)=\varphi (x)\;\mathrm{Sin}(2\pi m\frac{x-t\;v(t)}{l})$$

where φ(x)  is the waveform, *l* is the backbone length, and v(t)  is the relative wave velocity. As the backbone length is constant during bending, we have

(3) $$\begin{array}{l} \int_{x_{Ai}}^{x_{A(i+1)}}\left(\begin{array}{l} 1+(\frac{\partial \varphi }{\partial x}\mathrm{Sin}(2\pi m\frac{x-t\;v(t)}{l})+\\ \varphi (x)\;(\frac{2\pi m}{l})\mathrm{Cos}(2\pi m\frac{x-t\;v(t)}{l}){)}^{2} \end{array}\right)\;dx=l/6 \end{array}$$

This equation is numerically solved to obtain the position of *A_i_* points. Consequently, coordinates of *R_i_* points are obtained readily as

(4) $$x_{Ri}=x_{Ai}+(\hat{n}.\hat{i})d_{i}=x_{Ai}-d_{i}\frac{\partial f/\partial x}{\sqrt{1+\partial^{2}f/\partial x^{2}}}\left|\begin{array}{l} x=x_{Ai} \end{array}\right.\;y_{Ri}=y_{Ai}+d_{i}(\hat{n}.\hat{j})=y_{Ai}+\frac{d_{i}}{\sqrt{1+\partial^{2}f/\partial x^{2}}}\left|\begin{array}{l} x=x_{Ai} \end{array}\right.$$

Likewise, for the *L_i_* points, we will have

(5) $$x_{Li}=x_{Ai}+d_{i}\frac{\partial f/\partial x}{\sqrt{1+\partial^{2}f/\partial x^{2}}}\left|\begin{array}{l} x=x_{Ai} \end{array}\right.\;y_{Li}=y_{Ai}-\frac{d_{i}}{\sqrt{1+\partial^{2}f/\partial x^{2}}}\left|\begin{array}{l} x=x_{Ai} \end{array}\right.$$

Thus, the system states are obtained for *i* = 1..6 using

(6) $$q_{i}={\left({\left(x_{R(i+1)}-x_{Ri}\right)}^{2}+{\left(y_{R(i+1)}-y_{Ri}\right)}^{2}\right)}^{0.5}\;q_{i+6}={\left({\left(x_{L(i+1)}-x_{Li}\right)}^{2}+{\left(y_{L(i+1)}-y_{Li}\right)}^{2}\right)}^{0.5}$$

Finally, the state vector $\vec{q}$ is numerically measured for one cycle in *N* steps and is saved as a matrix $Q=\left[\left.{\vec{q}}_{1}\right|\left.{\vec{q}}_{2}\right|\left.{\vec{q}}_{3}\right|\left.\ldots \right|{\vec{q}}_{N}\right]$. Matrix Q is the desired actuators’ length corresponding to the desired anguilliform gait. The matrix can be normalized to represent contraction rates for the McKibben actuators. The ‘offline’ calculated matrix is repeatedly given to the robot actuators with the desired frequency to repeat the gait. (Note that the relation between the actuator length (or the contraction rate) and pressure is supposed to be linear. The maximum contraction (10 percent) corresponds to maximum pressure (7 MPa) and the interval values can be expressed with pulse width modulation (PWM), which is given as the input signal to the solenoid valves.

The kinematic equations can be used for different reasons, especially explanations of the robot’s working principles, and geometric design. The equations are used to demonstrate the robot’s performance before fabrication graphically. They can be used for optimization, e.g., maximizing the bending amplitude. Nevertheless, a desired kinematic may differ from the actual observation. In practice, the robot’s kinematic properties and motion arise from the interactions of modes and cannot be fully explained by considering the individual robot. Emergent phenomena often exhibit behavior or characteristics that are not predictable from the properties of the parts in isolation. On the other hand, control of a pneumatic system, i.e., air pressure control, is hard. The actual kinematics can be different from the desired kinematics due to random factors (such as leakage, fault, etc.) and the fluid interplay with the compliant body. Even the existence or dominance of a traveling wave within the robot swimming can be vailed under such circumstances. We propose data analysis to obtain insight into such phenomena.

### 2.2. Proposed CDE DMD Algorithm

Suppose the lateral position and velocity of *s* digitized points of the robot midline are recorded as $y\in {\mathbb{R}}^{s\times 1}$ and $v\in {\mathbb{R}}^{s\times 1}$ in discrete time intervals, Δt. A filtered velocity vector $\bar{v}$ is defined for the discrete signal v using a canonical discrete filter model

(7) $${\bar{v}}_{,k}≃\bar{v}[k]=\sum_{i=0}^{d-1}v[k-i].h[i]$$

where the filter kernel is chosen $h[i]=\frac{1}{d}$, d∈ℕ for a moving average filtration. The complex state vector at time t=kΔt is shown by $z_{,k}\in {\mathbb{C}}^{s\times 1}$ and is defined as

(8) $$z_{,k}=y_{,k}+j{\bar{v}}_{,k}$$

Note that in this context, the *i*th entry of a vector like $z_{,k}$ is denoted as $z_{i,k}$ (and similarly, for representing the *i*th entry over all the times, we use $z_{i,}$). The time-resolved matrix, $Z\in {\mathbb{C}}^{s\times n}$, is constructed with the complex state vectors as its columns, that is

(9) $$Z=\left[\begin{array}{ccc} \left.\right|\; & \left.\right|\; & \;\\ z_{,1} & z_{,2} & \\ \left.\right|\; & \left.\right|\; & \; \end{array}\begin{array}{ccc} & \; & \left.\right|\;\\ \cdots & & z_{,n}\\ & \; & \left.\right|\; \end{array}\right]$$

The time-delay embedded matrix $H\in {\mathbb{C}}^{s(d+1)\times (n-d)}$ with the embedding dimension number d is constructed as follows

(10) $$H=\left[\begin{array}{cccc} z_{,1+d} & z_{,2+d} & \ldots & z_{,n}\\ z_{,d} & z_{,1+d} & \ldots & z_{,n-1}\\ . & . & & .\\ . & . & \ldots & .\\ . & . & & .\\ z_{,1} & z_{,2} & \ldots & z_{,n-d} \end{array}\right]$$

Based on a linear approximation of the Koopman operator, the DMD approach assumes a linear mapping that approximates snapshots of the system dynamics one timestep forward. The DMD algorithm takes the matrices of snapshots

(11a) $$X=\left[\begin{array}{c} \;\left|\right.\\ h_{,1}\\ \;\left|\right. \end{array}\begin{array}{c} \;\left|\right.\\ h_{,2}\\ \;\left|\right. \end{array}\begin{array}{c} \ldots \end{array}\begin{array}{c} \;\left|\right.\\ h_{,n-d}\\ \;\left|\right. \end{array}\right],\;X^{+}=\left[\begin{array}{c} \;\left|\right.\\ h_{,2}\\ \;\left|\right. \end{array}\begin{array}{c} \;\left|\right.\\ h_{,3}\\ \;\left|\right. \end{array}\begin{array}{c} \ldots \end{array}\begin{array}{c} \;\left|\right.\\ h_{,n-d+1}\\ \;\left|\right. \end{array}\right]$$

where $h_{,k}$ represents column *k* of the H. It is supposed that a linear operator, as matrix $A\in {\mathbb{C}}^{s(d+1)\times s(d+1)}$, maps the column data to the next snapshot as in $h_{,k+1}=Ah_{,k}$. The best-fit solution relates the data matrices as

(11b) $$X^{+}=AX$$

Next, the method involves the SVD decomposition of the X matrix and order reduction, with truncation order *r* and matrices $U\in {\mathbb{C}}^{s(d+1)\times r}$, $\Sigma \in {\mathbb{C}}^{r\times r}$, and $W\in {\mathbb{C}}^{(n-d-1)\times r}$

(12) $$X=U\Sigma W^{*}=\left[\begin{array}{c} \;\left|\right.\\ u_{,1}\\ \;\left|\right. \end{array}\begin{array}{c} \;\left|\right.\\ u_{,2}\\ \;\left|\right. \end{array}\begin{array}{c} \ldots \end{array}\begin{array}{c} \;\left|\right.\\ u_{,r}\\ \;\left|\right. \end{array}\right]\left[\begin{array}{ccc} \sigma_{1} & & 0\\ & \ddots & \\ 0 & & \sigma_{r} \end{array}\right]{\left[\begin{array}{c} \;\left|\right.\\ w_{,1}\\ \;\left|\right. \end{array}\begin{array}{c} \;\left|\right.\\ w_{,2}\\ \;\left|\right. \end{array}\begin{array}{c} \ldots \end{array}\begin{array}{c} \;\left|\right.\\ w_{,r}\\ \;\left|\right. \end{array}\right]}^{*}$$

where $W^{*}$ represents the conjugate transpose of matrix W. Note that the reconstruction of the data is given by

(13) Xab=∑i=abu,iσiw¯i, , a=1 ,b=r

where ${\bar{w}}_{i,}$ represents the complex conjugate of vector $w_{,i}$. It follows that the DMD operator can be expressed as

(14) $$A=X^{+}D\;,\;D=W\Sigma^{-1}U^{*}$$

We can suppose a partitioning of D into DL and DN so that

(15) D=DL+DN

where

(16) DL=∑i=1kw,iσi−1u¯i, , DN=∑i=k+1rw,iσi−1u¯i,

Supposing DL contains selected modes with some properties of interest, in the linearity in this context, we can extract a AL matrix that captures the properties, using a partitioning A=AL+AN, so that

(17) AL=X+ DL

Then, an eigen decomposition AL=ΨΛΨ−1 is performed, where Ψ contains the eigenvectors as its columns and Λ is a diagonal matrix of the eigenvalues. Following an order reduction, $\Psi_{m}$ and $\Lambda_{m}$ are rewritten, keeping only *m* first eigenvectors and eigenvalues correspondingly. To obtain $f_{,k}$, the system state in the eigenbasis, $h_{,k}$, is transformed into a new coordinate system defined by the eigenvectors with the following relation $f_{,k}=\Psi_{m}^{-1}h_{,k}$. Then, in the modal space, we will have

(18) $$f_{,k+1}=\Psi_{m}\Lambda_{m}f_{,k}$$

Note that by transforming back to the original (delayed) basis, the solution can be represented as

(19) $$h_{,k}=\Psi_{m}\Lambda_{m}\Psi_{m}^{-1}h_{,0}$$

Finally, a matrix $\tilde{A}\in {\mathbb{C}}^{s\times s(d+1)}$ containing s rows of AL returns the predicted position data one timestep forward by the following relation

(20) $$y_{,k+1}\approx real(\tilde{A}h_{,k})$$

As a numerical method of discovering the patterns and underlying modes, the success of finding coherent structures depends, undeniably, on the physics of the system, in addition to the algorithm’s ability and the data’s quality and quantity. Therefore, simulation is an inseparable part of the method. The simulations and visualizations given in Section 3.2 are used to accomplish this exploration. In finding the delay embedding order, simulation results are intuitively useful. By increasing the number of delay coordinates, the embedding captures a larger portion of the system’s past dynamics, which in turn enhances the ability to reconstruct the underlying state-space representation. This is particularly useful for nonlinear systems with multi-scale behavior or chaotic dynamics. Nevertheless, excessive delay embedding might introduce redundant information if the system dynamics are inherently low-dimensional.

## 3. Results

### 3.1. The Eel Robot

**Figure 2:**
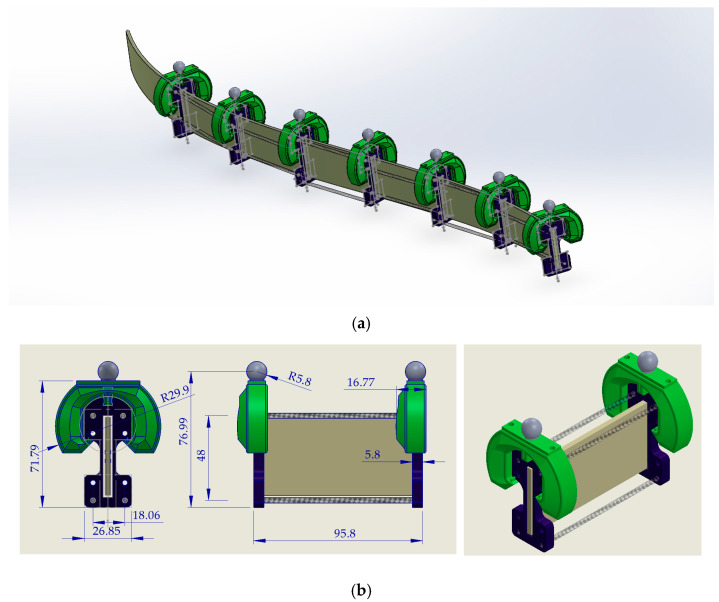
Mechanical drawings: (**a**) 3D model of the robot; (**b**) the segmental assembly parts and balancing bladders.

**Figure 3:**
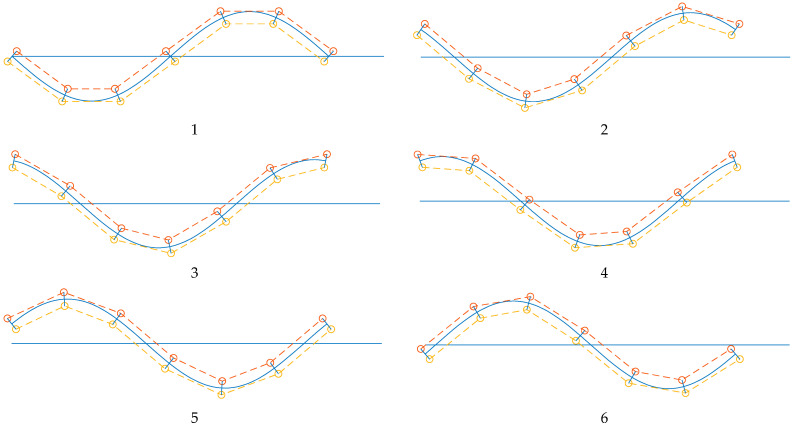
Geometrical representation of the desired actuator lengths calculations with a wave traveling left to right from 1 to 6. The blue curve represents the robot backbone axis versus the initial state, i.e., the straight blue line. The dashed lines represent the soft actuators’ axes.

Like flying propulsors, anguilliform swimmers such as eels and lampreys have flexible bodies bent by the contraction of lateral muscles. The muscles on the concave side are actively contracting in concentric mode, while those on the convex side exhibit reduced activity in the eccentric mode, forming antagonistic muscle systems. Inspired by this principle, we propose a soft structure, as shown in Figure 2. The robot contains a highly flexible beam with seven floats (the green components). The structure includes six segments that bend by two pairs of McKibben soft actuators. Seven markers (the gray balls in Figure 2) are used to track the backbone’s equally distanced points. The actuation geometry for steady swimming is simulated and graphically presented in Figure 3. Note that acceleration from rest, in contrast to steady swimming, can be associated with different kinematics, as reported about lampreys swimming [30]. Nevertheless, in this study, only a monotone input for steady swimming was applied to the robot from zero initial conditions.

**Figure 4:**
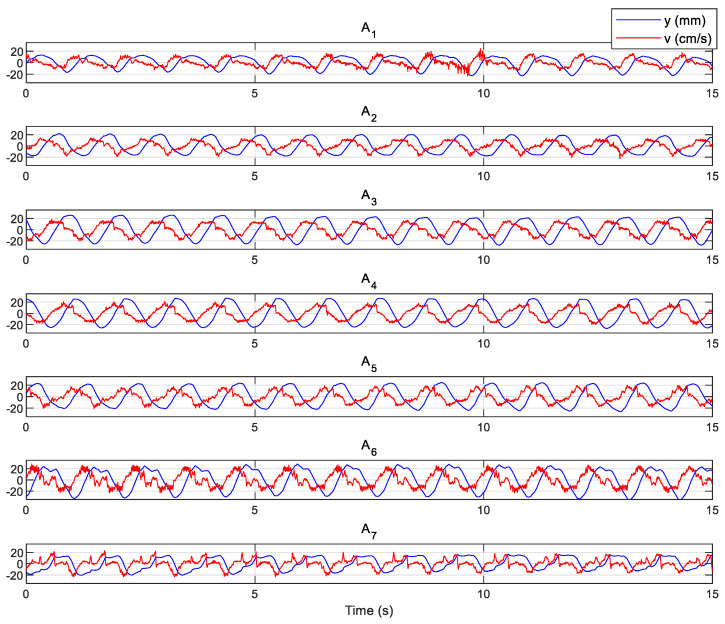
The experimental data. The robot’s undulation period is 1.1 s.

The graphs in Figure 3 present the MATLAB^®^ R2022b (MathWorks^®^, Natick, MA, USA) simulations of the equations and relations in Section 2.1. They can be used to verify the functionality principle, particularly the structure design’s compatibility. The dashed lines, representing the actuation axis, touch the ‘backbone’ at the maximum bending due to the opposite actuator. The minimum and maximum lengths of the dashed line are, respectively, equal to the length of an actuated muscle with the maximum pressure and the nominal length of an inactive muscle (as mentioned before, such a measurement can be performed within an optimization technique such as the genetic algorithm, which is not within the scope of this article). Nevertheless, the actual kinematics, i.e., the position and backbone curvature evolution, is highly influenced during a real swimming test. We are more interested in swimming kinematics and investigating the experimental data in this context. The experimental setup used for this purpose is explained in Appendix A. The data collected from a swimming test of the robot is illustrated in Figure 4, representing the tracking markers’ lateral displacement and velocities in the time domain. The velocities were calculated using differentiation of the measured position data. The time series plots are given for the seven tracking markers, *A*_1_ to *A*_7_. The visualization suggests a periodic motion with overall repetition expected from steady swimming.

A valuable tool for diagnosing mechanical behavior and analyzing vibratory systems is a phase plot. A phase plot is a graphical representation used in dynamical systems analysis to visualize the state of a system in phase space. It provides insights into the relationship between key dynamic variables, such as position and velocity when the system evolves in time. In the context of system dynamics, the phase plot is particularly useful for examining oscillatory or non-linear behavior, offering a concise way to understand the motion without explicitly referencing time. To construct a phase plot, one requires time-series data of the system’s position and velocity. The real axis represents the position, while the imaginary axis represents the velocity. By treating position as the horizontal component and velocity as the vertical component, the trajectory in the phase space is plotted as the system evolves. Each point on the curve corresponds to a unique state of the system at a particular moment in time. This method is especially effective for oscillatory systems, where periodic motion appears as closed loops, indicating energy conservation, while spiraling trajectories may suggest dissipative effects or damping.

**Figure 5:**
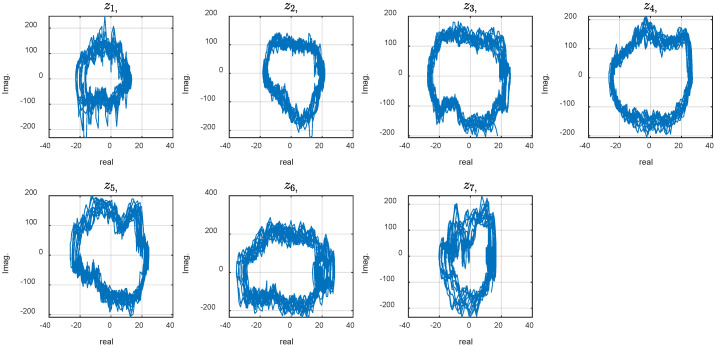
Phase plot of the experimental data.

The phase plot of the experimentally measured values is illustrated in Figure 5. The graph represents the complex states $z_{i,}$ for the 7 tracking markers (*i* = 1..7). The advantages of using a phase plot include its ability to highlight the system’s characteristics. It can visually reveal attractors, periodicity, or chaotic behavior. Unlike time-domain plots, which may obscure the interplay between variables, phase plots emphasize their relationship directly, enabling a more intuitive understanding of system dynamics. Nevertheless, the phase graphs of the experimental data do not illustrate coherent structures. At first look, it seems impossible to draw a deduction about the system dynamics or contribution of nonlinearity, stochastic, noise, or chaotic phenomena. In the CDE DMD, we look for a new coordinate system where coherent structures or patterns are revealed.

### 3.2. CDE DMD Analysis Results

**Figure 6:**
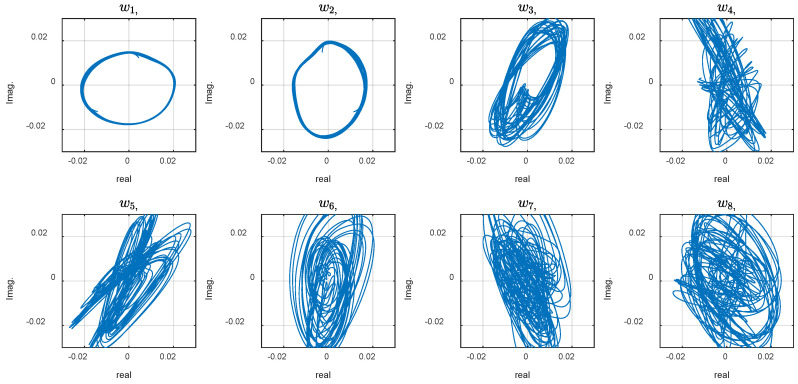
Phase plot of the dynamic variables with *d* = 30.

**Figure 7:**
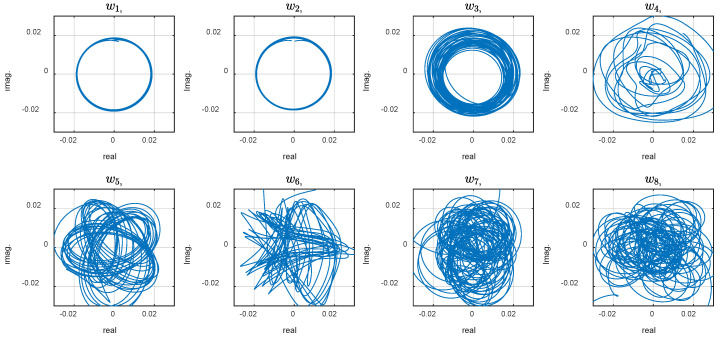
Phase plot of the dynamic variables with *d* = 110.

The phase plot reveals strange behavior of the system that, at first look, may be attributed to random, chaotic, or noisy phenomena within the robot or its interaction with the environment. We expect the phase plots to appear as perfect circular shapes for a perfectly swimming system exhibiting a smooth sinusoidal wave. On the other hand, with chaotic (but not random) systems, smooth trajectories where each point does not go back to its place in the previous period, resulting in repeating coherent curves, are anticipated. Previous studies attributed the exotic dynamic to the system modes are measured from the FSI model and simulations [7,31]. Such simulations are numerically expensive and need modeling assumptions and approximations. This study proposes the CDE DMD analysis described in the method to discover the underlying modes that may explain the system behavior based on the experimental data. For this aim, calculations with different embedding dimension, d, were conducted. The phase plot of the dynamic variables for d=30 and 110 are shown in Figure 6 and Figure 7. Increasing the delay steps yields more circular shapes, revealing coherent attractors in the leading modes in the CDE coordinates. Note that the sampling time was Δt=0.005 s, and the robot actuation period was T=1.1 s. Therefore, the delay number d=110 corresponds to a half cycle of the undulation.

**Figure 8:**
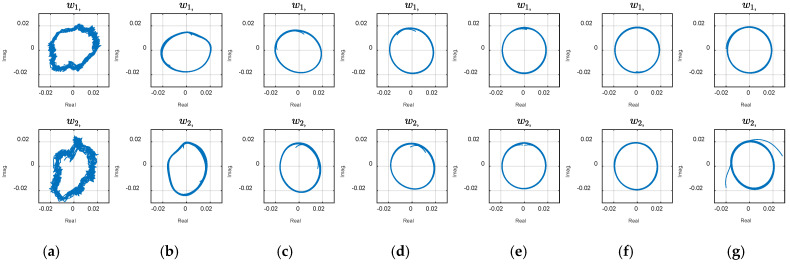
Evolution of the first two modes with embedding dimension: (**a**) Standard DMD; (**b**) CDE DMD with *d* = 30; (**c**) *d* = 60; (**d**) *d* = 85; (**e**) *d* = 110; (**f**) *d* = 140; (**g**) *d* = 170.

The circular graph of the first two variables, $w_{1,}$ and $w_{2,}$, in Figure 7 indicates the presence of two harmonic oscillations in the CDE coordinates. The phase plots represent the temporal modes that construct the overall dynamics. Coherent patterns and structures appear in the ‘CDE coordinate system’ (in contrast to the ‘physical coordinate system’ where the measurement is performed). Delay embedding contains some limitations, necessitating numerical assessment to verify the method for a specific application. High dimensionality, unknown delay number, and noise handling may be limitations, depending on the underlying physics of a particular application. Note that increasing the embedding dimensions by more delay steps increases the matrix size and unnecessary calculation complexity and noise. The right number of delay steps, which is often unknown, is estimated using simulations and observing the modes. The number is increased and the evolution of the first two modes is considered as an optimization criterion. As shown in Figure 8, by diverging from the optimal value (Figure 8e), the graphs start to show ovality.

**Figure 9:**
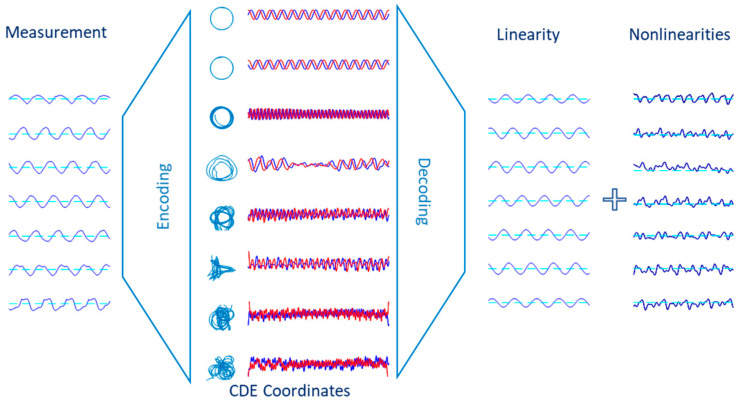
Conceptualization of the linearization technique. Measurement data are projected to the CDE coordinates, in the encoding stage. Consequently, the linear (and nonlinear) portion is obtained by superimposing the linear (and nonlinear chaotic) modes, in the decoding stage.

The working principle of the CDE DMD method is shown abstractly in Figure 9. The measured data, in the physical coordinate system, is first encoded by projecting the data to the CDE coordinates. Encoding involves increasing the dimensionality due to the large size of the Hankel matrix. Then, superimposing linear modes, in the decoding stage, provides the linear dynamics. Similarly, nonlinearities can be obtained by com-posing the nonlinear modes.

**Figure 10:**
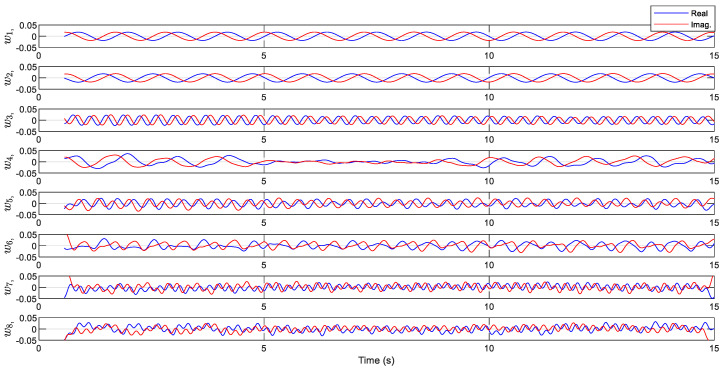
Time-series plot of the CDE dynamic variables with *d* = 110.

**Figure 11:**
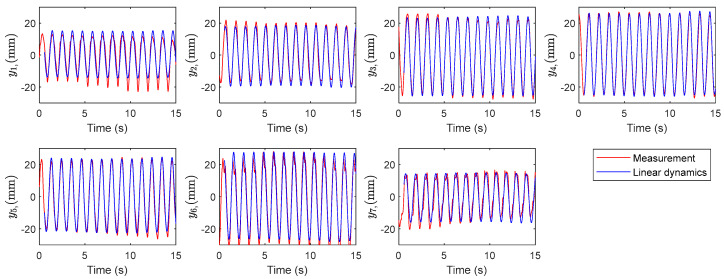
The linearized model versus the measured data.

**Figure 12:**
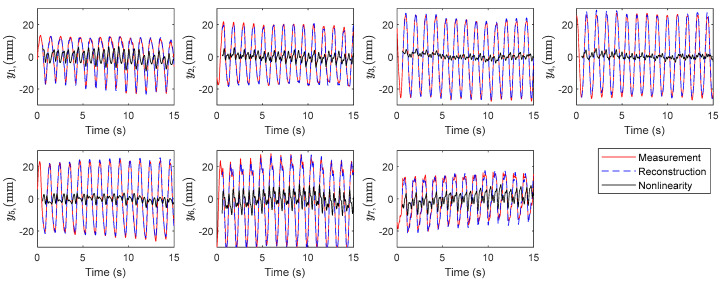
Comparison of the reconstructed data, the nonlinearity contribution, and the measurements.

The corresponding time history of the variables visualized in Figure 10 indicates the linearity property within the dominant modes. The first two modes present smooth oscillations with a constant amplitude typical in mass-spring and linear vibration systems. The phase shift, comparing mode one and mode two, is attributed to traveling wave phenomena. It is inferred from the results that the temporal modes consist of nearly linear oscillators disturbed by chaotic modes. This fact suggests partitioning the system dynamics into an approximate linear subsystem and a highly nonlinear chaotic subsystem. Therefore, the reconstruction of the model with the linear modes results in a linear surrogated system model. Reconstruction of the data with the linear and nonlinear subsystems is shown in Figure 11 and Figure 12. The linear dynamics, shown in Figure 11, is composed of the first two modes, corresponding to X12 in Equation (13). In Figure 12, the full reconstruction, from X112, as well as the nonlinearity portion, corresponding to X312, is demonstrated. The graphs contain the experimental data for comparison.

**Figure 13:**
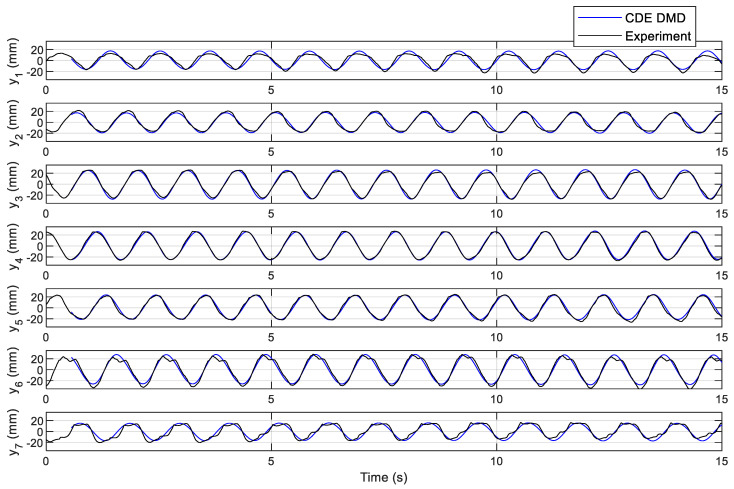
Comparison of the CDE DMD results with the original data.

Therefore, the CDE DMD provides a linearized model by including the first modes. The simulation results of the linearized model Equation (20) are shown in Figure 13. In the numerical calculations, *H* was a 777 by 2890 matrix with a rank of 777 calculated after the construction of the matrix. With 7 sensed points, *s* = 7, and 110 delays, *d* = 110, the number of rows of *H* became *s(d + 1)* = 777. Likewise, with the measurement sampling time of 0.005 s and a total time of 15 s, *n* = 15/0.005 = 3000, the number of columns will be equal to *n* − *d* = 2890. The AL and A matrices are of size 777 by 777, while $\tilde{A}$ is a 7 by 777 matrix.

### 3.3. CDE DMD’s Noise Handling

**Figure 14:**
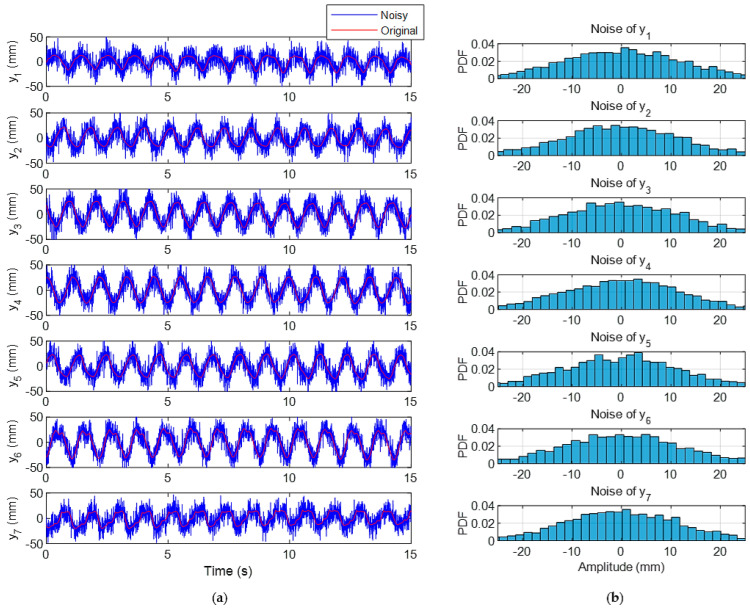
Data with injected noise: (**a**) The original and corrupted data; (**b**) Histogram of the added noise.

The CDE DMD handles noise effectively by leveraging delay embedding to distribute noise across dimensions, applying low-rank approximations via SVD and isolating dominant modes. These properties make it a robust tool for analyzing noisy measurements, particularly when the signal-to-noise ratio is moderate to high. In this Section, the ability of the method to handle noisy data is examined by simulation. As the measurement was achieved with a precise laboratory setup, a random signal was added to simulate the corruption of the original signal by the artificial noise. The noisy signal and the probability density function (PDF) of the added noise are shown in Figure 14. In this case, the noise contains Gaussian signals with a mean of zero and standard deviations of 12.

**Figure 15:**
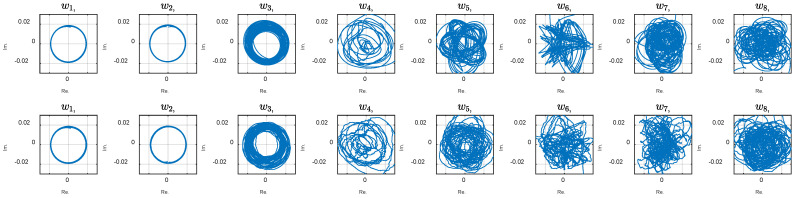
Comparison of the dynamic variables of the original (**top**) and the corrupted (**bottom**) data.

The influence of added noise on the dynamic variables, i.e., the CDE modes, is shown by simulation. Figure 15 illustrates the results for the original signal (top phase plots) and the corresponding modes for the noisy signal (bottom plots). A comparison of the plots shows that the first two modes remain similar, before and after the noise injection. In fact, the noise is projected to higher modes due to its high-frequency nature. 

It is also worth mentioning that random and chaotic signals are distinguishable in such graphs. Chaos exhibits repeating and smooth modes while random signals do not show such deterministic and predictable patterns.

**Figure 16:**
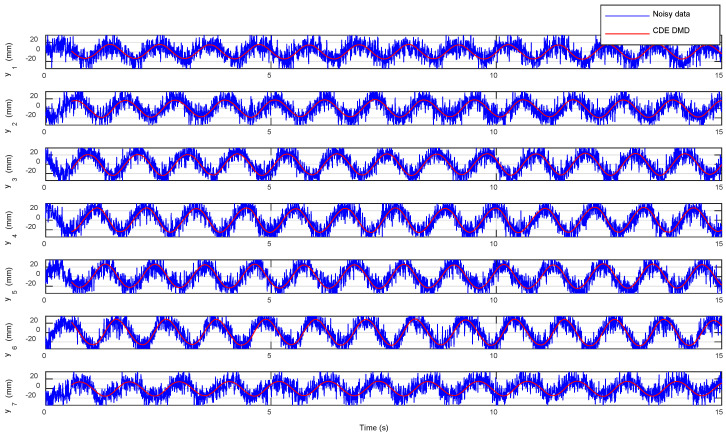
Comparison of the CDE DMD results with the noisy data.

The performance of the CDE DMD linearized model with the noisy data is shown in Figure 16. The results represent the ability of linearization in the presence of highly noisy data. This advantage can be useful with inherently noisy measurement systems, such as inertial measurement units (IMUs), or in-field measurements where extra environmental noise and vibrations are inevitable.

## 4. Discussion

The results of this study demonstrated that the experimental (raw) data reflect the robot’s compliance (i.e., influenceability by the external loads) but do not display smooth oscillations. As a side effect of compliance, the system is prone to deformations due to any external loads or faults within the system. Attributing such fluctuations to random or chaotic phenomena does not appear straightforward. The delay embedding results, on the other hand, reveal coherent structures and oscillators within the system dynamics. In particular, the phase plots reveal linear and chaotic influences that are typically observed in many fluid dynamic problems. Within the phase plot of the experimental data, coherent structures are not visible, and the interplay of noise or chaos is not discoverable. The delay embedding and recovering the model with the linear modes resulted in a linearized model of the system. The simulation results showed that the linearized model captures a large portion of the system dynamics, compared with the experimental results. Additionally, the method can separate the nonlinearity effect, which is useful in signal filtering.

From the phase lag within the time series plot of the first two modes, it is inferred that the modes construct the traveling wave along the robot body. This conclusion was drawn similarly in the previous research using intensive FSI simulations. The linearized model, introduced as the CDE DMD model, matches well with the experimental data. Such a model has different applications. The first goal of the linearization, in this study, was to investigate the existence of a traveling wave within the robot locomotion and explain the disorder. The linear model acts as a predictor that has many applications in robotics. Firstly, the predictor can be used for prediction purposes as in fault detection and outlier recognition. Faults in pneumatic actuators (leakage) and outliers (missed points during tracking) within measurements are common problems, which can be detected by comparison of the measured data with the predicted data. Secondly, the model is potentially useful for navigation (path following). Classic control deals with stabilization around zero, which is extended to control over a desired path. Similarly, the anguilliform swimmer oscillates along a curve (path), which is considered a straight line in the first step. The linear model works as a filter that can be used to eliminate nonlinearity effects. Another question that the results can explain, at least as a lateral conclusion, is related to the repeatability of the measurements. A classic question to be answered within any scientific measurement in general and for aquatic locomotion, in particular, is how the experimental observation is repeated in different runs. Chaotic and random signals are known to be notoriously unrepeatable. The results show that robot locomotion is mainly ruled by approximately linear dynamics disturbed by ‘small’ chaotic modes. This means that the gross motion of the robot is approximately predictable and repeatable, while the minor fluctuations are not. Simulations with added noise show the CDE DMD’s capability of handling excessive noise. The method is proposed to extract the main modes of body undulation, especially for soft robots, which are highly influenced by additional modes and environmental vibrations. Soft robots, in contrast to rigid robots, are prone to deformation due to water interaction. Application for other types of aquatic locomotion is to be investigated as future work, but it is imaginable that carangiform and thunniform swimming contain a single linear mode (compared to two linear modes in anguilliform swimming).

## 5. Conclusions

This paper investigates the kinematics of a soft eel robot actuated by pneumatic muscles. The experimental results of the swimming test were used as the raw data for further analysis using a DMD-based algorithm. The algorithm employs the delay embedding technique to cope with the limitation of measurement points, compared to the infinite dimensionality of the system. The simulations showed that coherent structures are revealed with sufficient embedding order (here, 110 lags is equivalent to a half period of the robot undulation) in the complex delay embedded (CDE) coordinate systems. In particular, two linear oscillators were detected, which were attributed to the traveling wave (anguilliform undulation). The two modes compose the linearized model, which captures the gross motion of the robot. Additionally, the nonlinearity is composed of chaotic modes. It is discussed that the CDE DMD provides a prediction model that can be used in various applications such as data filtering, fault detection, or navigation applications as the future works. The CDE DMD’s ability to analyze anguilliform locomotion characteristics in the presence of noise and body fluctuations makes it proper for studying soft-bodied swimmers, particularly soft robots.

## Figures and Tables

**Figure A1:**
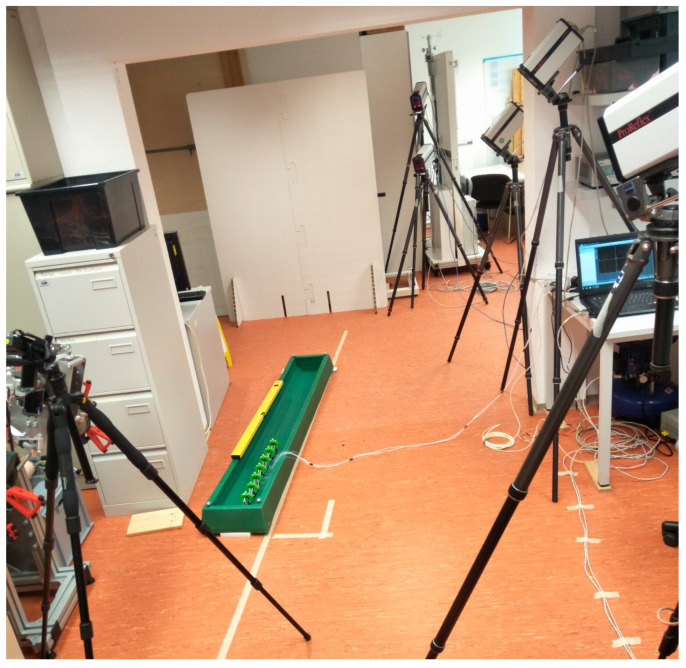
The measurement setup.

## Data Availability

The original contributions presented in the study are included in the article; further inquiries can be directed to the corresponding author.

## Appendix A

The experimental setup used in this research is shown in Figure A1. The measurement system contains a QualiSys^®^ Tracking Manager (QTM) 1.6.0.1, which collects the position of tracking markers in Δt=0.005 s intervals. The position data are saved as ‘.tsv’ files and are post-processed offline using Python 3.8.19 within the Spyder 5.5.1 integrated development environment and imported in MATLAB^®^ R2022b. The velocities were obtained by calculation (differentiation).
